# Rapid and Visual RPA-Cas12a Fluorescence Assay for Accurate Detection of Dermatophytes in Cats and Dogs

**DOI:** 10.3390/bios12080636

**Published:** 2022-08-13

**Authors:** Liyang Wang, Jinyu Fu, Guang Cai, Xiyu Cheng, Di Zhang, Shuobo Shi, Yueping Zhang

**Affiliations:** 1 College of Veterinary Medicine, China Agricultural University, 100193 Beijing, China; 2 Beijing Advanced Innovation Center for Soft Matter Science and Engineering, College of Life Science and Technology, Beijing University of Chemical Technology, Beijing 100029, China; 3 College of Life Sciences and Bioengineering, School of Physical Science and Engineering, Beijing Jiaotong University, Beijing 100000, China

**Keywords:** RPA-Cas12a, dermatophytes, *Microsporum canis*, *Trichophyton mentagrophytes*

## Abstract

Dermatophytosis, an infectious disease caused by several fungi, can affect the hair, nails, and/or superficial layers of the skin and is of global significance. The most common dermatophytes in cats and dogs are *Microsporum canis* and *Trichophyton mentagrophytes*. Wood’s lamp examination, microscopic identification, and fungal culture are the conventional clinical diagnostic methods, while PCR (Polymerase Chain Reaction) and qPCR (Quantitative PCR) are playing an increasingly important role in the identification of dermatophytes. However, none of these methods could be applied to point-of-care testing (POCT). The recent development of the CRISPR (Clustered Regularly Interspaced Short Palindromic Repeats) based diagnostic platform promises a rapid, accurate, and portable diagnostic tool. In this paper, we present a Cas12a-fluorescence assay to detect and differentiate the main dermatophytes in clinical samples with high specificity and sensitivity. The Cas12a-based assay was performed with a combination of recombinase polymerase amplification (RPA). The results could be directly visualized by naked eyes under blue light, and all tested samples were consistent with fungal culture and sequencing results. Compared with traditional methods, the RPA-Cas12a-fluorescence assay requires less time (about 30 min) and less complicated equipment, and the visual changes can be clearly observed with naked eyes, which is suitable for on-site clinical diagnosis.

## 1. Introduction

Dermatophytosis, an infectious disease caused by several fungi, can affect the hair, nails, and/or superficial layers of the skin and is of global significance [1]. The most common dermatophytes in cats and dogs are *Microsporum canis* (zoophilic) and *Trichophyton mentagrophytes* (zoophilic and anthropophilic) [2]. In cats, 98% of dermatophytosis cases are caused by *M. canis* [3]. Dermatophytosis is transmitted by animal-to-animal or animal-to-environment contact with infectious substances (i.e., spores, hyphae) [2]. Clinical appearances are highly variable, including hair loss, papules, erythema, scaling, crusting, and hyperpigmentation, with or without pruritus.

**Figure 1:**
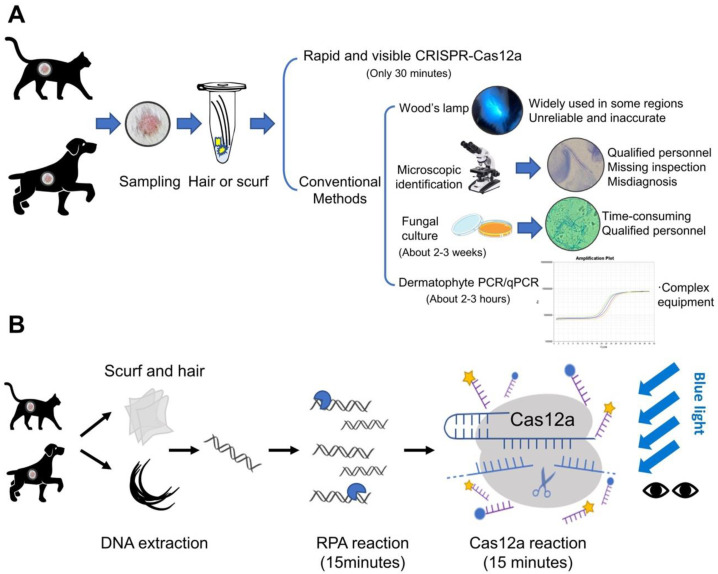
Schematic representation of diagnostic methods for dermatophytosis. (**A**) The methods of dermatophyte detection are summarized into two categories: The first category is CRISPR-Cas12a-based detection. It only takes 30 min after DNA extraction, with high specificity and sensitivity; The other is conventional detection methods including Wood’s lamp examination, microscopic identification, fungal culture and molecular detection methods (PCR and qPCR). (**B**) The Cas12a-based assay was performed in conjunction with recombinase polymerase amplification (RPA). After DNA extraction of scurf and hair samples, the tests are completed within 30 min. The results could be observed directly by naked eyes under blue light.

The clinical diagnostic methods for dermatophytosis include Wood’s lamp examination, microscopic identification, fungal culture, and molecular detection methods (PCR or qPCR) based on internal transcribed spacers (ITS) (Figure 1). Wood’s lamp examination is widely used in some regions for initial rapid detection of dermatophytosis, regardless of the unreliable and inaccurate nature of this technique. The microscopic cytology findings from clinic samples can help clinicians start initial treatment before the fungal culture results, however, this approach requires specialized training and is inaccurate. Fungal culture can provide reliable and accurate results, but the growth of the cultures usually takes about 2 weeks. Molecular tools, targeting ITS sequences, have been increasingly developed for dermatophytes detection [4] (including conventional PCR [5,6], real-time PCR [7], nested PCR [8], multiplex PCR [9], PCR enzyme-linked immunosorbent assay [10], and PCR-restricted fragment length polymorphism [11,12,13,14]). The ITS regions are nonfunctional DNA regions located between structural ribosomal RNAs (rRNAs), which are commonly used for fungal taxonomy. In addition, the ITS sequence analysis is currently considered the “gold standard” diagnostic method for dermatophytosis [4]. PCR-based detection methods have shown the potential to detect pathogens in mixed infections or environmental fungal contamination in approximately 2–3 h [15], but it is more dependent on available laboratory conditions and facilities [16]. Apart from the above methods, MALDI-TOF (matrix-assisted laser desorption/ionization time-of-flight) mass spectrometry (MS) has also been reported in dermatophytes identification [17], while it is limited by the database availability and facilities. Therefore, none of these methods could be applied to point-of-care testing (POCT). The rapid detection of dermatophytes allows veterinarians to implement antifungal treatment in time. In addition, the identification of specific dermatophytes can help veterinarians educate owners on effective prevention methods thereby effectively avoiding disease recurrence and potential human infections.

In recent years, CRISPR-Cas12a protein has received special attention in the field of molecular diagnostics. Like CRISPR-Cas9, the Cas12a protein has been used for genome editing for its RNA-guided dsDNA cleavage activity [18]. However, recent studies have also found that Cas12a protein has a target-activated, non-specific single-stranded deoxyribonuclease (ssDNase) cleavage activity [19]. Briefly, after crRNA is specifically hybridized with target dsDNA, the Cas12a is activated to have indiscriminate cleavage activity to degrade ssDNA or RNA. Therefore, this activity can be applied to detect target dsDNA with the help of a fluorophore quencher (FQ)-labeled ssDNA reporter (i.e., FAM which can be visualized with the naked eye under blue light). In addition, this technique is usually combined with an isothermal pre-amplification step for the target DNA enrichment, which requires less expensive equipment than PCR-based methods. For example, Chen et al. developed a rapid and accurate assay (DETECTR) for the detection and classification of human papillomavirus (HPV) in clinical specimens using Cas12a protein combined with recombinase polymerase amplification (RPA) [20]. Cas12a expands the range of diagnostic applications for infectious diseases, such as the detection of viruses (i.e., pandemic COVID-19 [21,22] and African swine fever virus [23]) and bacteria [24] (i.e., *Staphylococcus aureus* [25], *Listeria monocytogenes* [26], *Mycobacterium tuberculosis* [27], and Methicillin-resistant *Staphylococcus aureus* [28]), and it can also be used for pathogen detection in agricultural [29] and aquatic community [30]. Because CRISPR-Cas12a detection assay has been around for a short time, few studies have been done on fungi pathogens detection, especially dermatophytes. The recent research on fungal detection using Cas12a was for wheat fungal diseases [31]. Additionally, positive samples can also be identified by naked eyes in a relatively short period of time by this method. Based on such characteristics, CRISPR/Cas12a detection can be developed into a portable tool that is more helpful for clinical diagnosis in the future. In conclusion, CRISPR/Cas12a technology shows the potential for a rapid, accurate, and portable diagnostic tool [32].

It has been noted that small animal clinics need an accurate, rapid, and user-friendly detection method that addresses the shortcomings of traditional dermatophyte diagnostic methods. In this study, we established a method to detect and identify the two main species of dermatophytes using an RPA-Cas12a-fluorescence assay. Additionally, we demonstrated its great potential for point-of-care dermatophytosis diagnosis.

## 2. Materials and Methods

### 2.1. Sample Collection

We obtained a total of 31 clinical samples from cats and dogs from three animal clinics in China (29 samples with clinical signs and 2 healthy control cat samples as in Table 1). The samples with clinical signs were collected through hair plucked out from the edges of skin lesions or with apple-green fluorescence using Wood’s lamp, and scurf gathered from hair coats. The healthy cat samples were collected through hair plucked out from the healthy skin (sample 31 and 33). By observing different amounts of infectious materials under microscopy in the clinics, 28 patients were diagnosed with dermatophytosis, with sample No. 25 diagnosed as non-dermatophytosis. Through Wood’s lamp inspection on some clinical samples, a classic “apple-green” fluorescence could be observed on the hair coats of 22 patients (Table 1). We also collected a hair and nail sample from the experimenter as an operational control (sample 29) and a desk wipe sample of the animal clinic as an environment control (sample 30) (Table 1). We also collected a mouse skin tissue extract sample as another animal control (sample 34) and a pure water sample as a reaction control (sample 35).

### 2.2. Strains, Clinical Isolates and Fungal Culture Conditions

We used the standard dermatophyte strains from American Type Culture Collection: *T. mentagrophytes* ATCC 28185, and *M. canis* ATCC 32903. In the laboratory, standard strains and clinical samples were inoculated on the Sabouraud Dextrose Agar (SDA) plate containing 200 mg/L chloramphenicol and incubated at 30 °C for 2 weeks. The samples of all cases were identified by observing the macromorphology and micromorphology of fungal colonies after about 2 weeks, and then fungal samples were subjected to PCR amplification using ITS1 and ITS4 primer set [33] (Table 2). All the primers were synthesized by Sangon Biotech (Shanghai, China). The PCR products were submitted to Sangon Biotech for sequencing, and species were identified by NCBI Blast (https://blast.ncbi.nlm.nih.gov/Blast.cgi accessed on 30 July 2022).

### 2.3. DNA Extraction from Clinical Samples and Isolates

The hair and scurf samples were extracted by mixing in a 45 μL extraction buffer (50 mM sodium hydroxide (NaOH)) and incubated at 95 °C for 5 min, then neutralized by 5 μL of 1 M Tris-HCl, pH 8.0 buffer. After mixing, the samples were centrifuged at 12,000 rpm for 5 min, and then the supernatant was collected. This DNA-containing solution was prepared for the template for the RPA assays. The DNA from the fungal colonies (pieces of a colony of 3–5 mm diameter on plate) was extracted by the same procedures as above, and these samples were amplified by ITS primers and sequenced. The DNA concentration for *M. canis* extract is 564.4 ng/μL, and the DNA concentration for *T. mentagrophytes* extract is 342.1 ng/μL.

### 2.4. Generation of dsDNA Targets

The sequences used for RPA primers design were obtained from the NCBI Nucleotide Database (GenBank accession numbers MH858319.1 and NR_131265.1). Primers for RPA were listed in Table 2. RPA reactions were performed by the Twist-Amp basic kit (TwistDX, British). Each RPA reaction (50 µL) contained 29.5 µL rehydration buffer, 2.4 μL forward and reverse primers, 2 µL genome DNA extraction samples, 2.5 μL of 280 mM magnesium acetate (MgAc), and 11.2 μL water. The mixtures were incubated at 39 °C for 15 min. For gel analysis, the RPA products were cleaned up using 70% ethanol precipitation method and verified by electrophoresis on a 1% agarose gel. For Cas12a detection, the 1 μL of RPA products without purification were directly added to Cas12a reaction. For the limit of detection determination, we extracted genomic DNA from reference strains, quantified DNA concentration with NanoDrop 2000C (Thermo Fisher Scientific, Waltham, MA, USA), calculated copy-number/volume using dermatophyte genome sizes, and diluted DNA samples to as far as a single genome in RPA reactions.

### 2.5. Cas12a Expression and Purification

A his-tagged (C-terminal) codon-optimized version of Cas12a (*Francisella tularensis subsp. novicida*) gene was synthesized from Sangon Biotech (Shanghai, China). The expression plasmid (pET28a-FnCas12a) was transformed into BL21 (DE3), then, BL21 (DE3) cells carrying the expression plasmid were cultured in Luria-Bertani (LB) medium at 37 °C overnight. The cells were transferred into fresh LB (1:100 inoculation) at 37 °C until OD600 nm reached 0.8. Then, induced with 0.5 mM IPTG and transferred to 18 °C for 16-h expression. Cells were collected by centrifuged at 15,000 rpm for 15 min and resuspended in 50 mL of lysis buffer [50 mM Tris-HCl (pH 8.0), 1.5 M NaCl, 1 mM DTT and 5% glycerol] with 1 mM phenylmethanesulfonyl fluoride (PMSF) as the protease inhibitor and lysed by high pressure. Then, the lysis was centrifuged at 15,000× *g* for 30 min and the supernatant was loaded onto HisTrap HP column (GE Healthcare, Madison, WI, USA). The column was then washed with wash buffer (lysis buffer supplemented with 30 mM imidazole) and eluted with elution buffer (lysis buffer supplemented with 600 mM imidazole). The collected protein was dialyzed in storage buffer (20 mM Tris-HCl, pH 8.0, 600 mM NaCl, 1 mM DTT, 0.2 mM EDTA, 15% (*v*/*v*) glycerol) and finally stored in aliquots at −80 °C.

### 2.6. Transcription of crRNAs

The preparation of crRNA proceeded in three steps. The transcription templates for crRNA preparation were amplified by the PCR process, with the primers listed in Table 2. Then, the transcription process was performed at 37 °C overnight using the T7 High Yield Transcription Kit (Thermo Fisher Scientific, Waltham, MA, USA). Finally, the transcript products were purified using the RNA Clean & ConcentratorTM-5 (Zymo Research, Irvine, CA, USA) and quantified with NanoDrop 2000C (Thermo Fisher Scientific, Waltham, MA, USA).

### 2.7. Cas12a Detection

Cas12a cleavage reaction system is consisting of 500 nM Cas12a, 500 nM crRNA, 2 µL target DNA (RPA products from genomic DNA extract of standard strains or clinical samples), 500 nM ssDNA (FAM- GATCAAGAGCTA -BHQ1) and 0.5 µL RNase inhibitor (TaKaRa, Osaka, Japan) in a 50 µL volume. The reactions were performed at 37 °C in NEB buffer 3.1 for 15 min. The total 50 µL reaction products were first exposed for 50 ms under blue light at the default settings of the Azure C300 Gel Imager (Azure Biosystems, Dublin, CA, USA). All 50 µL of reaction products were added to the 96-well plates and examined by Spire Multimode Plate Reader (PerkinElmer, Waltham, MA, USA). Then, 10 μL of the reaction was diluted 20 times to 200 μL and examined again by the plate reader. Another 10 μL of the products (without dilution) were placed under a Blu-ray glue cutter UV-Cut108 (LIFE iLAB BIO, Shanghai, China) and taken photos by a OnePlus 9R (mobile phone) in the default setting.

### 2.8. Statistical Analysis

Data are presented descriptively as mean average with standard deviation (SD) with triplicates. Statistical analysis was performed using a one-way ANOVA test and only significant (*p* < 0.001) values were marked with asterisks (***).

## 3. Results

### 3.1. Design and Detection of crRNA Guides and Primers in CRISPR-Cas12a Assay

**Figure 2:**
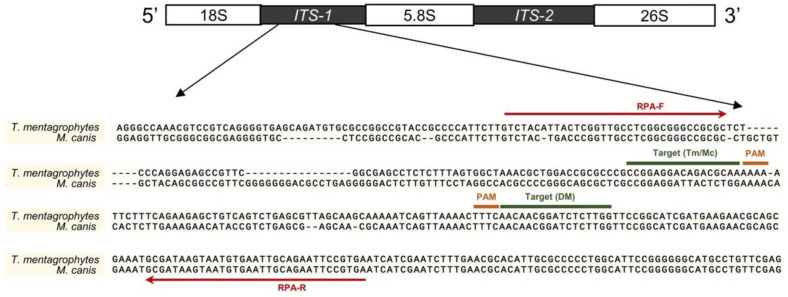
Primer and crRNA locations in the ITS region. Red lines indicated the primers of RPA; target sites of the crRNAs were marked in the corresponding area. Sequences were aligned using MUSCLE (SnapGene software 4.0, GSL Biotech LLC, San Diego, CA, USA) and illustrated by PowerPoint.

Because internal transcribed spacer (ITS) regions are considered the “gold standard” for identifying the fungi species, we designed the RPA-Cas12a assay by aligning the ITS sequences of *M. canis* and *T. mentagrophytes* (Figure 2). A common sequence was selected for crRNA guide recognition of dermatophytes (crRNA-DM) (this crRNA can also detect other dermatophytes, such as *Nannizzia gypsea*) and two specific sequences were selected for crRNA guide recognition of two main dermatophytes in cats and dogs: crRNA-Mc (*M. canis*) and crRNA-Tm (*T. mentagrophytes*) (Figure 2).

**Figure 3:**
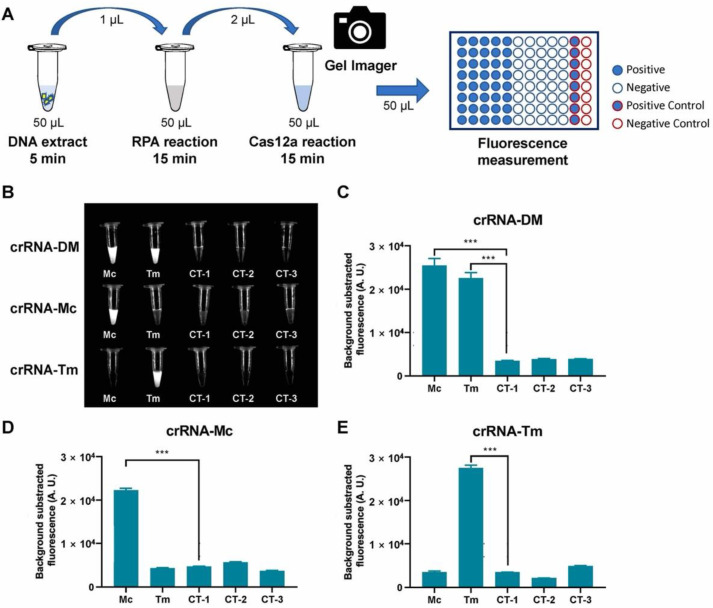
RPA-Cas12a fluorescence assay for reference strains. (**A**) Schematic representation of RPA-Cas12a fluorescence assay for reference strains. (**B**) Tube images of RPA-Cas12a fluorescence assay of dermatophytes (DM), *M. canis* (Mc), and *T. mentagrophytes* (Tm). CT-1, CT-2, and CT-3 were negative controls (Table 1). (**C**–**E**). Fluorescence detection of RPA-Cas12a fluorescence assay. The bar values represent the average of *n*  = 3 technical replicates, bars represent mean  ±  SD, statistical analysis was performed using one-way ANOVA test and significant values at *p* < 0.001 were marked with an asterisk (***).

To set up the detection assay, we carried out the RPA reaction using the DNA extract from two reference strains and three negative controls, then added the RPA products directly to the Cas12a fluorescence assay (Figure 3A). To examine the visual fluorescence signals using the same exposure time, we took a photo of all testing tubes under blue light using a gel imager (Figure 3B). The results showed that the crRNA-DM can detect two reference strains and two specific crRNAs can identify corresponding dermatophytes with obvious signals compared to control groups. To measure the fluorescence signals, three replicates of each Cas12a reaction were measured by the plate reader, and the results were consistent with the tube images by gel imager (Figure 3B–E). The fluorescence signals of the targeted samples are significantly different (*p* < 0.001) from negative controls and non-targeted samples.

### 3.2. Sensitivity of the RPA-Cas12a Fluorescence Assays

**Figure 4:**
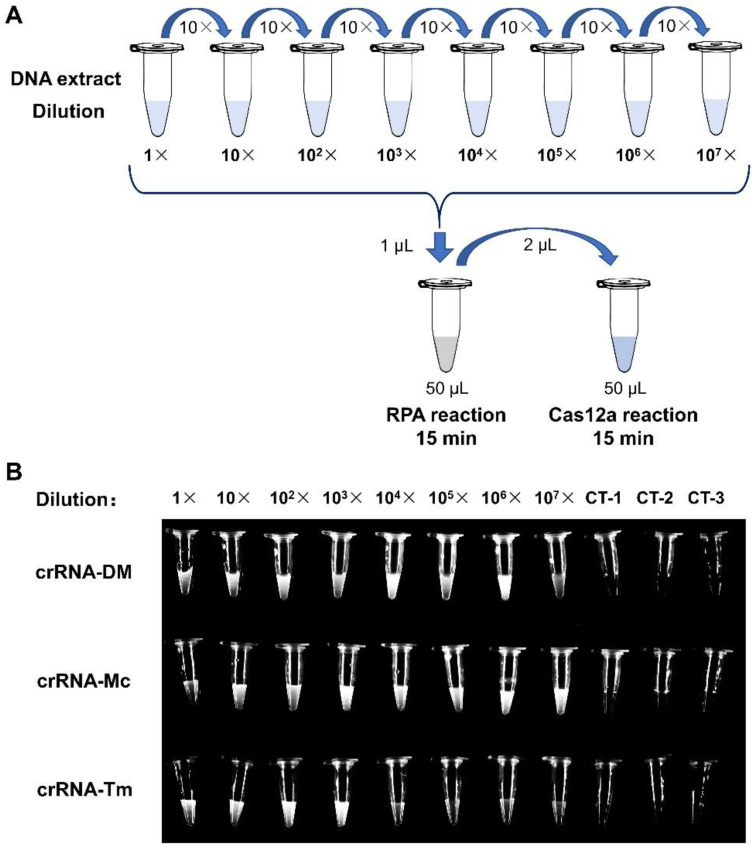
The Sensitivity of the RPA-Cas12a fluorescence assays. (**A**) Schematic representation of RPA-Cas12a fluorescence sensitivity assay. (**B**) Tube images of RPA-Cas12a fluorescence sensitivity assay. For crRNA-DM and crRNA-Mc assays, we used the RPA products of *M. canis* reference strain; and for crRNA-Tm assays, we applied the RPA products of *T. mentagrophytes* reference strain. CT-1, CT-2, and CT-3 were negative controls (Table 1).

In order to determine the sensitivity of the RPA-Cas12a fluorescence reporting assays, we generated serial dilutions (1×, 10^1^×, 10^2^×, 10^3^×, 10^4^×, 10^5^×, 10^6^× and 10^7^×) of the DNA extracts from reference strains and performed RPA-Cas12a fluorescence assays, respectively (Figure 4A). The imaging results showed that the fluorescence signals of crRNA-DM (1 to 10^6^×), crRNA-Mc (1 to 10^7^×), and crRNA-Tm (1 to 10^3^×) were dramatically stronger than the negative control and can be distinguished by naked eyes under blue light (Figure 4B).

### 3.3. The Specificity and Sensitivity of RPA-Cas12a Fluorescence Assay for Clinical Samples

We collected 29 clinical samples and 6 controls for RPA-Cas12a fluorescence assay verification. The clinical diagnosis of dermatophytosis was based on clinical signs, Wood’s lamp examination and direct microscopic identification by the clinic doctors (Table 1). A total of 29 samples had different degrees of clinical signs, and microbe infections can be found in 28 samples under the microscope.

**Figure 5:**
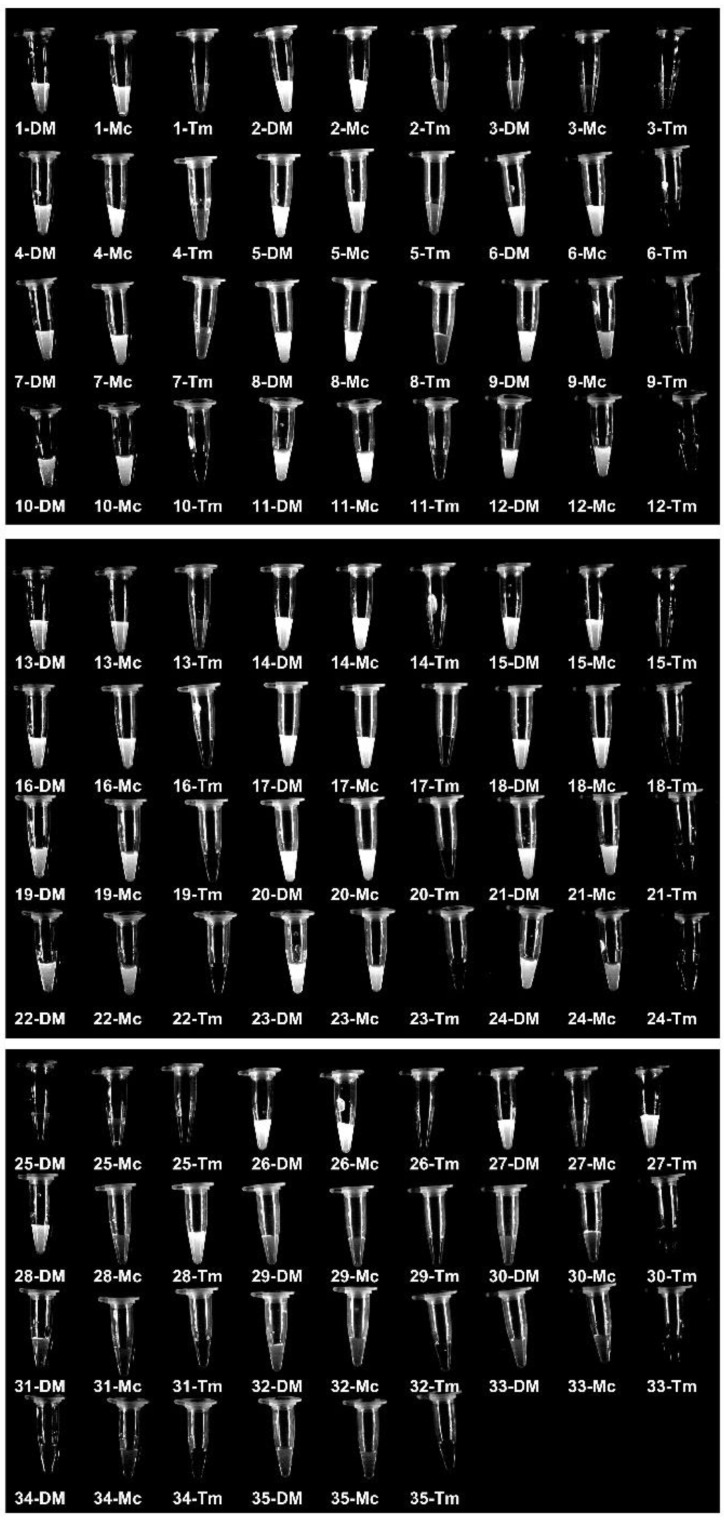
RPA-Cas12a fluorescence assays of clinical samples. Tube images of RPA-Cas12a fluorescence detection of 35 samples using guide crRNA-DM (DM), crRNA-Mc (Mc) and crRNA-Tm (Tm), respectively. The samples were described in Table 1.

**Figure 6:**
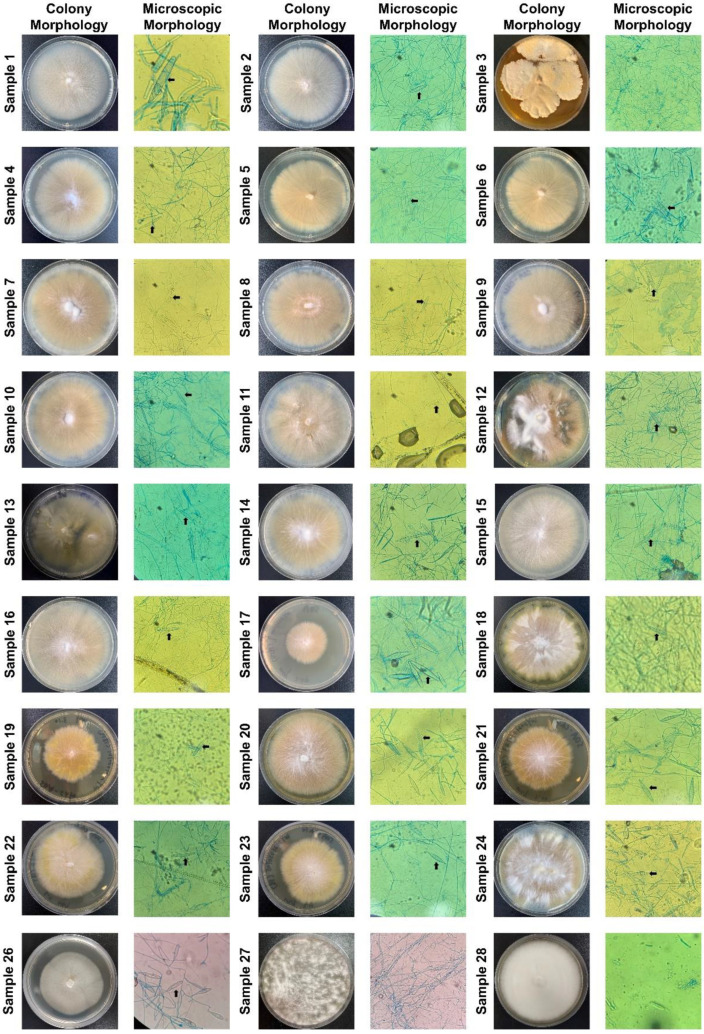
Fungal cultures and microscopic identification of clinical samples. All clinic samples were inoculated on the SDA plate and grown for two weeks. We only showed the plate and microscopy images of colony growth. The black arrows pointed to spindle-shaped macroconidia in 24 plates (400× magnification), indicating the presence of *M. canis*.

We used the RPA-Cas12a fluorescence assay to detect these clinical samples (directly using hair and scurf) so that we can determine the specificity and sensitivity. The results showed that 26 samples were identified as dermatophytes, 24 samples were identified as *M. canis*, and 2 samples were identified as *T. mentagrophytes* (Figure 5). Meanwhile, we inoculated all samples on SDA plates and incubated them at 30 °C for 2 weeks. After colony growth, we performed micromorphological studies and ITS sequencing for these clinic isolates (Figure 6 and Table 1). *M. canis* produces septate hyphae and spindle-shaped macroconidia, and the macroconidia can be identified in all *M. canis* positive samples (black arrows in Figure 6). While samples No. 27 and 28 were negative for Wood’s lamp examination, while our RPA-Cas12a fluorescence assay tests were positive for *T. mentagrophytes* which were consistent with microscopic morphology. The sequencing results were searched using NCBI Blast to identify microorganisms. The ITS sequencing results of the culture samples were considered standard results (Table 1). The sequencing results all agreed with the results of RPA-Cas12a fluorescence assay.

We were initially surprised about the results for sample No. 3, as it was initially diagnosed with dermatophytosis in clinics (screened out a few spores under the microscope in clinics). The results of ITS sequencing showed that the main colony of sample No. 3 in the medium was identified as *Chaetomium globosum*, which is a saprophytic fungus, that primarily resides on plants, soil, straw and dung, which has not been shown as a pathogen. Based on the negative result of the culture, we suspected that the saprophytic fungi spores observed under the microscope may be carried by the animal patient, and this caused the misdiagnosis. The clinical signs may be caused by other reasons in the patient while *Chaetomium globosum* could be the result of environmental contamination.

There is another case, the negative result of RPA-Cas12a fluorescence in sample No. 25 was consistent with the results of fungal culture and clinical diagnosis (Table 1). This patient showed focal hair loss which was similar to the signs of dermatophytosis while the clinical diagnosis result was non-dermatophytosis (Table 1). To further determine the specificity of our method, we attempted to collect other types of samples, for example, we tested another kind of fungi pathogen *Malassezia* (Sample No. 32), control samples from healthy cats (Sample No. 31 and 33), a hair and nail sample from the experimenter (Sample No. 29), and an examination desk wipe sample from an animal clinic as the environment control (Sample No. 30). Additionally, all these controls gave negative results of RPA-Cas12a fluorescence and fungal culture.

The results showed that our RPA-Cas12a fluorescence assay also successfully and specifically detected *M. canis* and *T. mentagrophytes* in these clinic samples, and more importantly, the results of control samples were all negative. Importantly, the Cas12a fluorescence results were consistent with laboratory diagnostic results from colony morphology, microscopic morphology and ITS sequencing (Figure 6 and Table 1). In our limited testing, the RPA-Cas12a method could achieve 100% sensitivity and 100% specificity.

## 4. Discussion

At present, the identification of dermatophytes in small animals still relies on comprehensive diagnostic methods, such as Wood’s lamp examination and microscopy in clinical practice. Some clinics would perform fungal culture and dermatophyte PCR for further diagnosis. The diagnosis methods currently used in clinical practice have their own limitations (Figure 1A). For example, the diagnosis by clinic signs sometimes may mislead veterinarians. Due to the variability of the lesions, pruritus, and other lesions may occur in different animals. It also can be confused with other diseases such as pemphigus foliaceus. Additionally, it has been reported that for Wood’s lamp examination, only 50% of *M. canis* infections can be detected, while most other dermatophytes (such as *T. mentagrophytes*) do not produce fluorescence [34]. Another common diagnostic method in clinical practice is a direct microscopic examination, which could be a simple and rapid way to screen for microconidia and hyphae, however, only >85% of cases can be accurately diagnosed [3]. Importantly, the professional requirements for the microscopic examination have high professional requirements. In addition, time-consuming and demanding (about 1–2 weeks) are two recognized disadvantages of fungal culture, which can delay the diagnostic outcome and treatment [3]. Molecular tools are increasingly used in the laboratory for fungal identification. Dermatophyte PCR or qPCR is becoming more and more popular due to its sensitivity, but it is not intuitive and more dependent on available laboratory conditions and the facilities, and the processing time is about 2–3 h. Taken together, these can cause confusion and inaccuracy [2]. In this case, accurate and timely identification of fungal isolates is very important. For clinical practice, developing new methods with rapid, intuitive, and highly specific identification of dermatophytes is crucial. We developed an RPA-Cas12a detection method for dermatophytes, which has shown excellent results in our research. The results were 100% consistent with the traditional fungal culture and ITS sequencing. The DNA extract can be diluted to more than 10^3^×, which shows that the assay had high sensitivity. All the positive samples can be sorted and visualized with naked eyes within 30 min at a constant temperature, which took less time than other molecular methods. For furthermore application in clinics, we hope to prepare an RPA-Cas12a quick detection kit for clinical use. The operator can collect animal hair or scurf for detection in about 30 min and visualize the results by naked eyes under the blue light.

In summary, the method we designed can assist veterinarians in accurately diagnosing two main dermatophytosis, especially when the operators are not yet proficient in the microscopic examination. In our study, we only used 29 samples with clinical signs and 6 control samples for validation. Therefore, more specimens should be included in the experiment to evaluate the effectiveness in the future.

## 5. Conclusions

In conclusion, the RPA-Cas12a-fluorescence assay is a promising method for detecting dermatophytes with high sensitivity and specificity. This method can rapidly detect the dermatophyte genome from clinical samples on site, and the detection time of dermatophytosis can be reduced by replacing the veterinarian with dermatophytosis. Therefore, the RPA-Cas12a fluorescence assay will be an excellent choice for point-of-care dermatophytosis diagnosis.

## Figures and Tables

**Table 1:** Clinical information and results of cases in the study.

No.	Type	Breed	Clinical Signs	Wood’s Lamp Examination	Microscopic Identification on Clinic Samples	Microscopic Identification on Culture Samples	ITS Sequencing on Culture Samples	RPA-Cas12a Detection on Clinic Samples
1	Cat	DSH	Alopecia and scurf	Positive	Conidium	*M. canis*	*M. canis*	*M. canis*
2	Cat	ASH	Crust	Positive	Conidium	*M. canis*	*M. canis*	*M. canis*
3	Cat	ASH	Alopecia, pruritus, and trauma	Not tested ^†^	Conidium	Undetermined *	*Chaetomium globosum*	Negative
4	Cat	DSH	Alopecia	Positive	Conidium	*M. canis*	*M. canis*	*M. canis*
5	Dog	Border Collie	Alopecia, pruritus, and trauma	Not tested ^†^	Conidium	*M. canis*	*M. canis*	*M. canis*
6	Cat	BSH	Alopecia and scurf	Positive	Conidium	*M. canis*	*M. canis*	*M. canis*
7	Cat	BSH	Alopecia and pruritus	Not tested ^†^	Conidium	*M. canis*	*M. canis*	*M. canis*
8	Cat	ASH	Alopecia and scurf	Positive	Conidium	*M. canis*	*M. canis*	*M. canis*
9	Cat	ASH	Alopecia and scurf	Positive	Conidium	*M. canis*	*M. canis*	*M. canis*
10	Cat	BSH	Alopecia and scurf	Positive	Conidium	*M. canis*	*M. canis*	*M. canis*
11	Cat	BSH	Alopecia and scurf	Positive	Conidium	*M. canis*	*M. canis*	*M. canis*
12	Cat	BSH	Alopecia and scurf	Positive	Conidium	*M. canis*	*M. canis*	*M. canis*
13	Cat	BSH	Alopecia and scurf	Positive	Conidium	*M. canis*	*M. canis*	*M. canis*
14	Cat	DSH	Alopecia	Positive	Conidium	*M. canis*	*M. canis*	*M. canis*
15	Cat	BSH	Alopecia and scurf	Positive	Conidium	*M. canis*	*M. canis*	*M. canis*
16	Cat	BSH	Alopecia and pruritus	Positive	Conidium	*M. canis*	*M. canis*	*M. canis*
17	Cat	Ragdoll	Alopecia and scurf	Positive	Conidium	*M. canis*	*M. canis*	*M. canis*
18	Cat	BSH	Alopecia, scurf, and erythema	Positive	Conidium	*M. canis*	*M. canis*	*M. canis*
19	Cat	Ragdoll	Alopecia	Positive	Conidium	*M. canis*	*M. canis*	*M. canis*
20	Cat	Russian Blue	Alopecia	Positive	Conidium	*M. canis*	*M. canis*	*M. canis*
21	Cat	Chinchi-lla	Alopecia	Positive	Conidium	*M. canis*	*M. canis*	*M. canis*
22	Cat	Persian	Alopecia	Positive	Conidium	*M. canis*	*M. canis*	*M. canis*
23	Cat	Persian	Alopecia and crust	Positive	Conidium	*M. canis*	*M. canis*	*M. canis*
24	Cat	DSH	Alopecia and scurf	Positive	Conidium	*M. canis*	*M. canis*	*M. canis*
25	Cat	ASH	Alopecia	Negative	-	No colony	-	Negative
26	Cat	Ragdoll	Alopecia	Positive	Conidium	*M. canis*	*M. canis*	*M. canis*
27	Cat	DSH	Alopecia	Negative	Conidium	*T. mentagrophytes*	*T. mentagrophytes*	*T. mentagrophytes*
28	Cat	DSH	Alopecia	Negative	Conidium	*T. mentagrophytes*	*T. mentagrophytes*	*T. mentagrophytes*
29	Experimenter	-	Control	Not tested ^†^	-	No colony	-	Negative
30	Environment	-	Control	Not tested ^†^	-	No colony	-	Negative
31	Cat	DSH	Healthy (Control)	Not tested ^†^	-	No colony	-	Negative
32	Cat	DSH	Pruritus	Not tested ^†^	*Malassezia*	*Malassezia*	*Malassezia pachydermatis*	Negative
33	Cat (CT-1)	ASH	Healthy (Control)	Not tested ^†^	-	No colony	-	Negative
34	Mouse (CT-2)	-	Healthy (Control)	Not tested ^†^	-	No colony	-	-
35	Pure water (CT-3)	-	Control	-	-	*-*	-	-

Note: DSH: Domestic Short Hair; ASH: America Short Hair; BSH: British Short Hair; Undetermined *: Miscellaneous colonies and no typical dermatophytes colonies; Not tested ^†^: One animal clinic did not perform Wood’s lamp examination. -: Not tested or no information.

**Table 2:** The primer sequences used in this study.

Method	Name	Sequences (5′-3′)	Molar Extinction Coefficients * (L/(mole·cm))	Products
PCR	ITS1	TCCGTAGGTGAACCTGCGG	179,300	ITS region
PCR	ITS4	GCATATCAATAAGCGGAGGA	211,800	
RPA	RPA-F	GTCTACATTACTCGGTTGCCTCGGCGGGCCGCGC	301,400	ITS-1
RPA	RPA-R	TCACGGAATTCTGCAATTCACATTACTTATCG	303,700	
crRNA	crRNA-R	GAAATTAATACGACTCACTATAGGG	258,700	
crRNA	crRNA-DM-F	CCAAGAGATCCGTTGTTATCTACAACAGTAGAAATTCCCTATAGTGAGTCGTATTAATTTC	603,400	Paired with crRNA-R for synthesizing crRNA-DM
crRNA	crRNA-Tm-F	CCGGAGGACAGACGCAAATCTACAACAGTAGAAATTCCCTATAGTGAGTCGTATTAATTTC	611,100	Paired with crRNA-R for synthesizing crRNA-Tm
crRNA	crRNA-Ng-F	CCGCCGGAGGAGTGATTATCTACAACAGTAGAAATTCCCTATAGTGAGTCGTATTAATTTC	604,100	Paired with crRNA-R for synthesizing crRNA-Ng
crRNA	crRNA-Mc-F	CCGGAGGATTACTCTGGATCTACAACAGTAGAAATTCCCTATAGTGAGTCGTATTAATTTC	601,100	Paired with crRNA-R for synthesizing crRNA-Mc

Note *: The molar extinction coefficient is calculated by OligoAnalyzer (https://www.idtdna.com/calc/analyzer accessed on 25 July 2022).

## Data Availability

Data are available upon request.

## Supplementary Materials

The following supporting information can be downloaded at: https://www.mdpi.com/article/10.3390/bios12080636/s1, Figure S1: The tube images of RPA-Cas12a fluorescence assay for reference strains using camera of a mobile phone. For visual detection, 10 μL of the RPA-Cas12a fluorescence assay from Figure 3B were placed under a Blu-ray glue cutter UV-Cut108 and taken photos by a OnePlus 9R (mobile phone); Figure S2: The tube images of the RPA-Cas12a fluorescence assays of samples using camera of a mobile phone. For visual detection, 10 μL of RPA-Cas12a fluorescence assays of samples were used for visual detection of a mobile phone camera.
