# Supplementary material for: Rapid and Visual RPA-Cas12a Fluorescence Assay for Accurate Detection of Dermatophytes in Cats and Dogs
Source: Biosensors (Basel). 2022 Aug 13;12(8):636. doi: 10.3390/bios12080636 (PMC9406134; doi:10.3390/bios12080636)
Supplement: Supplementary file 1 [file biosensors-12-00636-s001.zip › biosensors-1821410-supplementary.pdf]

Supplementary Figures

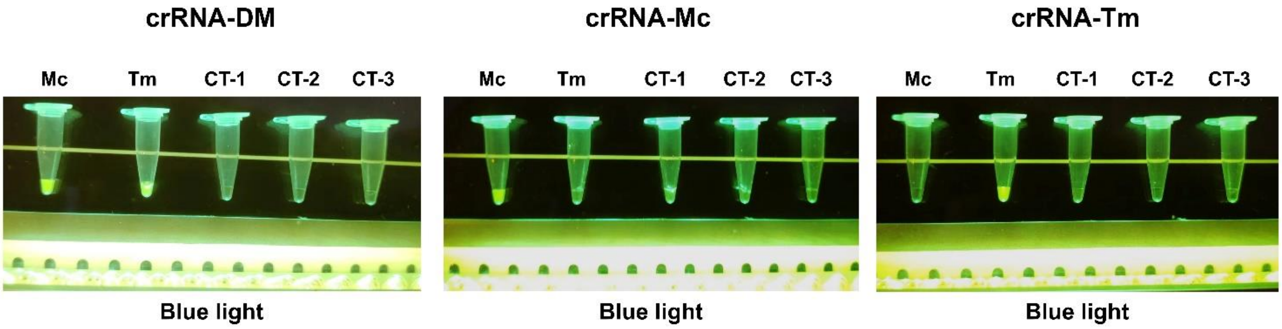

Supplementary Figure S1. The tube images of RPA-Cas12a fluorescence assay for reference strains using a mobile phone camera. For visual detection, 10 μL of the RPA-Cas12a fluorescence assay from Fig. 3B were placed under a Blu-ray glue cutter UV-Cut108 and taken photos by a OnePlus 9R (mobile phone).

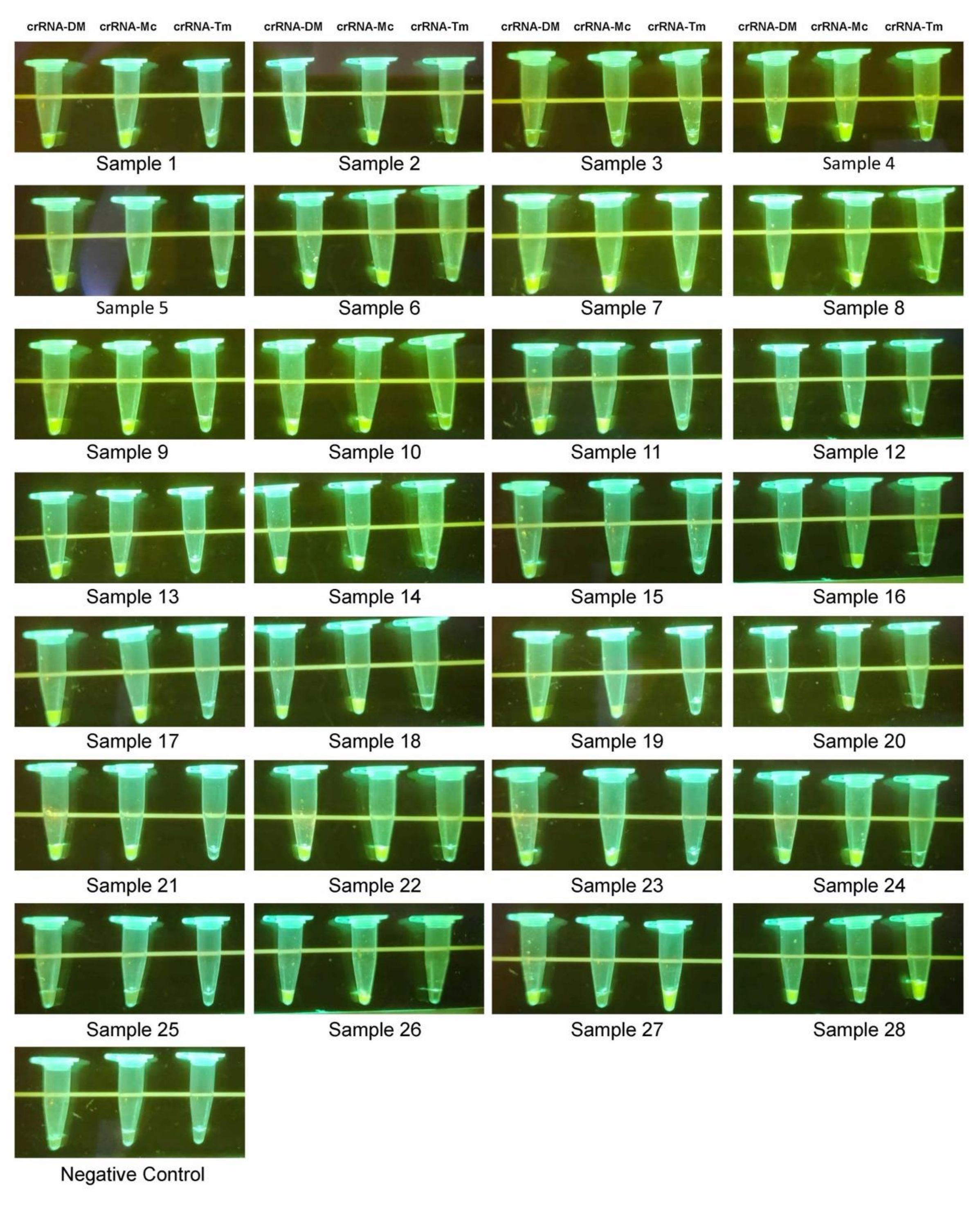

Supplementary Figure S2. The tube images of the RPA-Cas12a fluorescence assays of samples using a mobile phone camera. For visual detection, 10 μL of RPA-Cas12a fluorescence assays of samples were used for visual detection of a mobile phone camera.
